# Crystal-Growth-Controlled Exciton Funneling in BA_2_MAPb_2_I_7_ Ruddlesden–Popper Perovskite Thin Films

**DOI:** 10.3390/molecules31152636

**Published:** 2026-07-29

**Authors:** Grace Dansoa Tabi, Diego Florio, Chiara Botta, Alexandra J. Ramadan, Tersilla Virgili

**Affiliations:** 1 School of Mathematical and Physical Sciences, University of Sheffield, Hounsfield Road, Sheffield S3 7RH, UK; g.d.tabi@sheffield.ac.uk; 2 Istituto di Fotonica e Nanotecnologia—National Research Council (CNR), Piazza Leonardo da Vinci 32, 20133 Milan, Italy; diego.florio@polimi.it; 3 Dipartimento di Fisica, Politecnico di Milano, Piazza Leonardo da Vinci 32, 20133 Milan, Italy; 4 Istituto di Scienze e Tecnologie Chimiche “Giulio Natta”—National Research Council (CNR), Via A. Corti 12, 20133 Milan, Italy; chiara.botta@scitec.cnr.it

**Keywords:** quasi-2D Ruddlesden–Popper perovskites, transient spectroscopy, crystal growth, energy transfer

## Abstract

We investigate BA_2_MAPb_2_I_7_ (BAMA) quasi-2D Ruddlesden–Popper perovskite thin films prepared through single-crystal-derived and conventional polycrystalline routes. Morphological and X-ray diffraction analyses reveal significant differences in film texture, crystallinity, and phase distribution. Steady-state and time-resolved optical spectroscopies show that polycrystalline films are mainly composed of *n* = 2 and *n* = 3 phases and exhibit limited interphase energy transfer. In contrast, single-crystal-derived films display a richer excitonic landscape characterized by the presence of higher-(n) domains. Transient photoluminescence and pump–probe measurements demonstrate that the *n* = 2 exciton acts as the primary donor state and, uniquely in the single-crystal-derived films, undergoes two distinct transfer processes on the same timescale. The correlation between the decay of the *n* = 2 exciton and the population of lower-energy excitonic states provides direct evidence of hierarchical exciton funneling. These findings highlight the crucial role of phase distribution and crystallinity in governing exciton migration and energy-transfer pathways in low-dimensional perovskite heterostructures.

## 1. Introduction

Layered metal halide perovskite semiconductors are a versatile class of semiconductor materials, distinguished by their chemical and structural tunability [1,2,3,4,5,6]. Their structure comprises layers of inorganic metal halide octahedra cleaved by bulky organic spacer cations, which disrupt the characteristic three-dimensional (3D) perovskite structure [1,2,5]. This induces a quantum-well structure [2,3,6] in which the inorganic sheets provide the electronically active component [1,2,5], and the organic layers define the interlayer coupling [7,8,9], lattice distortion [7,10,11], quantum and dielectric confinement [3,6,12,13]. Through judicious choice of spacer cation chemistry, layered perovskites can be directed towards different structural families, including Ruddlesden-Popper (RP) [1,2,14,15,16], Dion-Jacobson [16,17], Alternating Cation Interlayer [18,19] and related layered motifs [7,8,12,16,17,20]. The chemistry of the spacer cation controls the length, rigidity, steric volume, polarity, and hydrogen-bonding character of the organic layer [7,8,9,10,13,21,22,23]. Through these parameters, the spacer cation shapes the electronic structure, carrier dynamics and photophysics of layered perovskites [7,8,9,13,21,24,25,26,27,28,29].

The number of metal halide octahedral sheets, denoted by *n* and hereafter referred to as the phase, and the chemistry of the organic and inorganic components of the perovskite can be selected to control the optical and electronic properties of the perovskite [1,2,12,14,15,21]. In the Ruddlesden Popper structure, increasing *n* increases the inorganic quantum well and progressively reduces quantum and dielectric confinement, generally lowering the bandgap and moving the electronic structure towards that of the 3D parent perovskite [6,12,14,15,24,25,26]. At small *n*, the inorganic sheets are strongly confined by the organic spacers, producing sharp excitonic absorption features [3,14,15,24], large exciton binding energies [3,6,13,24,27] and optical responses that are highly sensitive to local structural distortion [10,12,25,26,28,29]. These attributes have made layered perovskites attractive for optoelectronic devices where excitonic effects [24,27,28,29,30,31], radiative recombination [27,30,31,32], environmental stability [33,34,35] and structural anisotropy can be tuned [30,34,35,36].

For layered thin films with *n* > 1, achieving kinetic and thermodynamic control over the phase formation is challenging [37,38,39,40]. Quasi-2D perovskite thin films often manifest as a polydisperse distribution of phases, rather than a “phase pure” system [37,38,39,41,42,43,44]. This phase heterogeneity creates an energetic landscape of multiple bandgaps, which can strongly influence optoelectronic performance [32,36,38,41,42,45]. In such films, the phase that dominates absorption is not necessarily the phase that governs emission. Lower-*n* domains absorb at higher energies [14,15,32,37,38], while higher-*n* domains provide lower-energy states into which excitons or carriers can relax before recombination [32,36,38,41,45]. This energy funneling can be beneficial in light-emitting devices, where excitations are directed into emissive domains [45,46,47,48]. In photovoltaic or charge-transporting films, however, the same heterogeneity can introduce energetic disorder [21,42], band-tail states [21,42], trap-assisted recombination [38,39,40,43,44,49,50] and poor carrier transport [39,40,49]. Thus, a nominal *n* = 2 film should not be assumed to behave as an optically pure *n* = 2 semiconductor [37,38,41,42]. Its photophysics is determined by the phases that form [37,38,42], the phases that emit [32,36,41,45] and the energetic pathways between them [32,36,38,41,45,51].

As such, considerable efforts have been made to develop synthesis and processing strategies to enable control over the phase distribution of quasi-2D perovskite films [37,38,39,40,43,44,49,50,52,53]. At the most fundamental level, this control begins in the precursor solution. The relative amounts of spacer cation [7,8,9,21], small A-site cation [14,15,38], metal halide [1,2,14,15] and coordinating species [52,53] determine which metal-halide complexes form, how long they persist and which layered phases nucleate during drying [37,38,52,53]. In quasi-2D systems, phase selection is not determined by stoichiometry alone. The organic spacer and the small A-site cation do not necessarily incorporate into the lattice at the same rate during film formation [37,38,53]. Hence, the conversion process of iodoplumbate complexes can redirect crystallization towards competing phases, highlighting the need for additional strategies to regulate phase evolution [37,38,52,53]. Additives provide one such route to control the crystallization pathway [43,44,49,50,54,55,56]. They can modify precursor coordination [52,53,54], nucleation rate [38,49,53], defect formation and lattice relaxation [43,44,49,50], passivate under-coordinated lead or iodide sites [43,44,50,54], suppress phase disproportionation [43,44] and influence the orientation of the inorganic sheets [40,49,52,55,56]. A related strategy involves utilizing single-crystal-derived precursors to guide film formation, with recent work showing that this seed-assisted crystallization improves phase purity in BA_2_MA_2_Pb_3_I_10_ quasi-2D perovskite films and devices [37]. Solvent choice is equally important in determining how well precursors are dissolved and the rate at which the film moves from a solvated state into an ordered layered film [50,53,57]. Strongly coordinating solvents can stabilize lead-halide complexes and delay nucleation [53,57], while volatile or weakly coordinating solvent components can drive rapid crystallization and kinetic trapping [38,53,57]. These effects can decide whether a film crystallizes as a polydisperse distribution of phases or as a narrower and more coherent layered structure [37,38,43,44,50,52,53]. The subsequent processing conditions, including concentration [38,53], substrate temperature [38,40], spin speed [40,49], antisolvent timing [38,49], drying rate [38,53] and annealing protocol, can influence nucleation density, grain growth, texture, film thickness and residual strain [29,37,38,40,49,53]. In layered perovskites, crystallographic orientation is particularly important. Recent studies have demonstrated that such processing conditions strongly influence crystallographic orientation, crystallization kinetics, phase distribution, and carrier transport pathways in layered Ruddlesden–Popper perovskites [58,59,60,61]. When the inorganic slabs lie predominantly parallel to the substrate, out-of-plane carrier transport can be restricted by the insulating organic spacers [30,39,40,49]. More vertically oriented domains can provide more favorable pathways for carrier extraction [39,40,49,55], but they may also be accompanied by strain, mixed texture and additional structural heterogeneity [40,49,55,56].

Ultimately the photophysics of layered perovskites: exciton binding [3,6,13,24,27], spectral broadening [27,28,51], energy funneling [32,36,38,41,45], radiative recombination [27,30,31,32], non-radiative loss [38,39,40,41,42,43,44,49] and the apparent Stokes shift [24,27,28,51] all depend on how structural phases [9,14,15,23,37], morphology [37,40,49], texture [40,49,55,56] and energetic disorder [21,42,51] are coupled within the film. It is therefore important that we understand the influence that the chosen processing route has on the structural and hence photophysical properties of layered perovskites [37,38,39,40,41,42,49]. Different works [42,45,62,63] have been dedicated to understanding how to control the energy funneling between different domains in layered perovskites.

In this work, we investigate BA_2_MAPb_2_I_7_ (BA = butylammonium, MA = methylammonium, BAMA) quasi-2D Ruddlesden–Popper perovskite thin films prepared via single-crystal-derived and conventional polycrystalline routes. By combining morphological and X-ray diffraction analyses with steady-state and time-resolved optical spectroscopies, we demonstrate that the growth route strongly influences the phase distribution and energy-funneling dynamics in these films. In particular, the single-crystal-derived films exhibit an increased population of higher-*n* phases (*n* ≥ 3), leading to more efficient interphase exciton funneling toward lower-bandgap domains.

## 2. Results and Discussion

First, UV-Vis absorption spectroscopy was employed to characterize the optical properties of the quasi-2D Ruddlesden-Popper (RP) perovskite thin films. These quasi-2D RP crystal structures are represented by the formula (BA)_2_(X)*_n_*_−1_Pb_n_I_3*n*+1_, where *n* = 2; X = MA (methylammonium) [14,15]. For clarity, BA_2_MAPb_2_I_7_ is denoted as BAMA.

**Figure 1:**
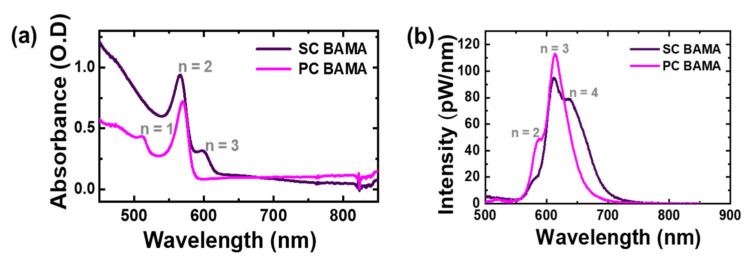
(**a**) UV-Vis and (**b**) photoluminescence (PL) spectra of single-crystal-derived BAMA and polycrystalline BAMA of the quasi-2D RP perovskite thin films spun on quartz substrates.

Figure 1a and Table S1 (see Supplementary Materials) present the UV-Vis absorption data measured for all investigated quasi-2D RP perovskite films. In Figure 1a, the UV-Vis absorption spectra of the BAMA series are dominated by strong excitonic peaks, a result of the high binding energy characteristic of these systems [3,6,7,10,14,15,24,27]. For single-crystal BAMA, a dominant excitonic absorption appears at 567 nm, consistent with the expected *n* = 2 exciton. In addition, a weaker feature at 600 nm is observed, attributable to trace *n* = 3 species, a phenomenon previously observed in similar RP systems [7,14,37,55]. In the polycrystalline BAMA sample, the primary peak shifts slightly to 569 nm, and a smaller peak appears at a higher energy (512 nm), which is characteristic of residual *n* = 1 phase contributions. The absorption data indicate that the two routes retain the main excitonic peak (*n* = 2) but show evidence of different minority phases.

Steady-state photoluminescence (PL) spectroscopy was used to investigate the emission properties of these quasi-2D RP perovskite systems. As displayed in Figure 1b, we observed that multiple phases with different “*n*” layer numbers coexist. The polycrystalline BAMA film exhibits a PL peak ranging from 545 to 680 nm with a shoulder at 585 nm (*n* = 2) and a dominant peak near 614 nm assigned to *n* = 3 emission. However, the single-crystal-derived BAMA film shows an even broader emission range, extending up to 728 nm. The spectrum contains contributions near 612 nm and 641 nm, assigned to *n* = 3 and *n* = 4 emissions, respectively. The enhanced broadening in the single-crystal-derived film suggests that the single-crystal growth route promotes the incorporation of higher-*n* inclusion, as observed in the absorbance [14,15,37]. This difference between the dominant absorption phase and the dominant emissive phase is central to the photophysics and carrier dynamics of these BAMA films. Excitons or carriers generated in high-energy, low-*n* domains migrate and relax into lower-energy, higher-*n* domains before radiative recombination [32,36,38,41,45].

**Figure 2:**
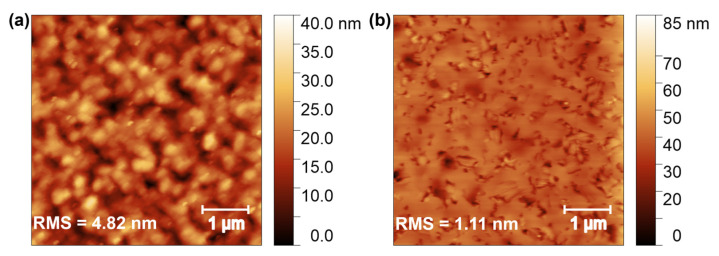
Representative atomic force microscopy images at a scan size of 5 µm for (**a**) single-crystal-derived BAMA and (**b**) polycrystalline BAMA of the quasi-2D RP perovskite thin films spun on quartz substrates.

Atomic force microscopy (AFM) was used to investigate the morphological differences between single-crystal-derived and polycrystalline BAMA films, presented in Figure 2. All films form compact, pinhole-free layers irrespective of fabrication route. Despite being prepared via a single-crystal-derived route, the resulting films consist of nanometer-scale crystallites rather than a single continuous crystalline domain, as presented in Figure 2a. The single-crystal-derived BAMA film exhibits a comparatively weakly textured surface, with no clearly resolved lateral features. In contrast, as seen in Figure 2b, the polycrystalline BAMA film shows a more pronounced platelet-like feature with a wide distribution of lateral sizes and shapes, indicative of a heterogeneous surface. Quantitative AFM analysis revealed that the root mean square (RMS) surface roughness was 4.82 nm for the single-crystal-derived BAMA film and 1.11 nm for the polycrystalline BAMA film, indicating higher roughness in the former. The increased roughness of the single-crystal BAMA film directly correlates with the broader diffraction patterns and additional crystallographic reflections observed in the X-ray diffraction measurements.

**Figure 3:**
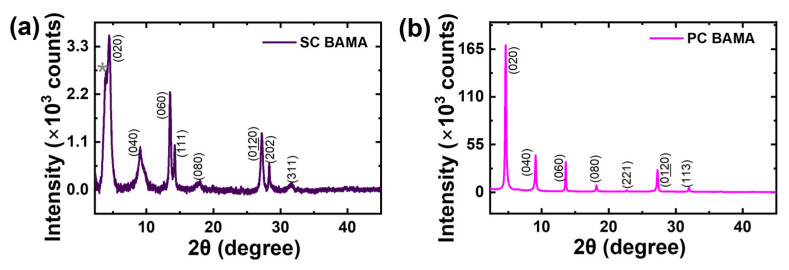
X-ray diffraction patterns of (**a**) single-crystal-derived BAMA and (**b**) polycrystalline BAMA of the quasi-2D RP thin film perovskites spun on quartz substrates. The asterisk is a non-identified phase.

Furthermore, the crystal structure and preferential orientation of the quasi-2D RP perovskite thin films were investigated using X-ray diffraction. In layered RP perovskites, increasing the layer index, *n*, corresponds to the systematic incorporation of additional corner-sharing PbI_6_ octahedral layers along with interleaved small A-site cations (MA^+^). This results in thicker inorganic slabs and corresponding changes in diffraction signatures [14,25,56,57]. The simulated XRD pattern for BAMA exhibits a characteristic (020) peak at 4.49°, obtained from CCDC 1478376. The dominant experimental diffraction peaks appear at 4.45° for single-crystal BAMA and 4.60° for polycrystalline BAMA, as shown in Figure 3a and Figure 3b, respectively. The deviations from the simulated positions may be indicative of lattice strain or microstrains induced during film processing. The single-crystal BAMA exhibits a broadening of the XRD peaks compared to its polycrystalline counterpart, suggesting a decrease in grain size. It is noteworthy that all peaks shifted toward lower 2θ angles in the single-crystal-derived thin films compared to their polycrystalline counterparts (Figure S1).

For both BAMA films, we observe an evenly spaced (0k0) family of planes, *n* = 2, indicating that the layered perovskite framework remains preferentially oriented with the inorganic sheets parallel to the substrate [9,14]. Additional reflections further distinguish between the two BAMA films. The single-crystal-derived BAMA film shows (111), (202) and (311) peaks near 2θ =14.2°, 28.3° and 31.7° respectively. In contrast, the polycrystalline BAMA only exhibits a (113) peak near 2θ = 32.0°. Nevertheless, magnified diffraction patterns in Figure S2 reveal weak (002), (111), (223) and (440) reflections near 2θ = 6.4°, 14.2°, 36.7° and 41.4° respectively. The weak low-angle reflection (6.4°) associated with *n* = 1 phase in Figure S2 is consistent with the *n* = 1 excitonic peak in the UV-Vis spectrum, see Figure 1a. The presence of peaks near 2θ = 14° and 28° may suggest vertical orientation of the PbI_6_ octahedral layers in the single-crystal-derived BAMA films [40,56]. The XRD data reinforce that despite the same nominal composition, the synthetic route defines the phase formation, preferred orientation and strain.

The observed differences in phase distribution are consistent with the seed-assisted crystallization mechanism reported by Sidhik et al. [37]. Their work showed that precursor solutions prepared from dissolved bulk layered perovskite crystals can retain local structural motifs, or “memory seeds”, which subsequently influence nucleation and crystal growth during film formation.

In agreement with this mechanism, dissolving pre-synthesized BA_2_MAPb_2_I_7_ single-crystals results in a broader distribution of layered phases, including higher-*n* domains. In contrast, the conventionally prepared polycrystalline precursor solution crystallizes through direct assembly of solvated precursor species, resulting in a narrower phase distribution. These structural differences are reflected in the XRD patterns and photoluminescence spectra. These phase distribution variations underpin the different exciton-transfer dynamics observed in the transient absorption and time-resolved emission spectroscopy measurements.

Film thickness measurements revealed average film thicknesses of 229 nm and 225 nm for the single-crystal-derived and polycrystalline BAMA films, respectively. The negligible thickness difference suggests that the distinct optical and structural properties observed for both BAMA films arise primarily from differences in crystallization pathway, phase distribution and surface morphology rather than variations in film thickness.

To explore the photophysics of those two films, we have performed femtosecond transient absorption (TA) spectroscopy. The measurements are discussed in terms of the fractional change in the transmitted probe signal

(1) $$\frac{\Delta T}{T}\left(t,\lambda \right)=\frac{T_{ON}\left(t,\lambda \right)-T_{OFF}\left(\lambda \right)}{T_{OFF}\left(\lambda \right)}$$

where $T_{ON}\left(t,\lambda \right)$ and $T_{OFF}\left(\lambda \right)$ are the transmitted ($T\left(\lambda \right)$) probe signals at a specific wavelength with and without the optical pump, respectively, and t is the pump-probe delay. All samples were photoexcited at 400 nm using ≈100 fs pulses with a fluence of ≈3 uJ cm^−2^.

**Figure 4:**
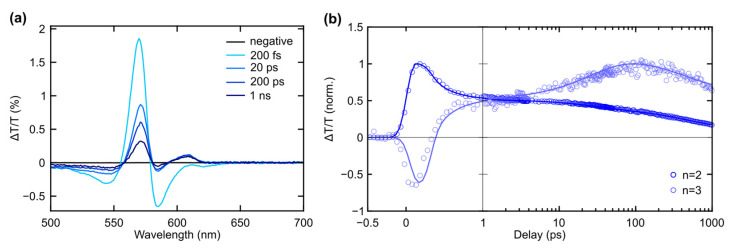
(**a**) Transient Absorption (TA) spectra at different pump-probe delays of the polycrystalline BAMA film. (**b**) TA dynamics of the polycrystalline BAMA film (open circles) at 570 nm (*n* = 2) and 610 nm (*n* = 3). The global fits are shown as solid lines. For clarity, the time axis is displayed on a linear scale up to 1 ps and on a logarithmic scale for longer delay times.

Figure 4a shows the pump-probe spectra at different pump-probe delays for the polycrystalline BAMA solid-state film. The spectra reveal the instantaneous formation of three photoinduced bands at 540 nm, 570 nm and 590 nm. The photoexcitation induces an instantaneous bleaching signal at 570 nm and two photoinduced absorption bands at 540 nm and at 590 nm, attributed to the bleaching of the *n* = 2 exciton and its photoinduced absorption to higher singlet states, respectively. The positive signal at 610 nm instead exhibits a long rise time of approximately 100 ps, and it is attributed to the combined contributions of bleaching and stimulated emission from the *n* = 3 exciton.

Figure 4b shows the dynamics of the TA signals at 570 nm and 610 nm. Following an initial rapid decay, likely due to hot-carrier cooling [64], the *n* = 2 exciton exhibits a slower decay component that closely matches the rise in the *n* = 3 excitonic signal. This process is attributed to energy transfer from the *n* = 2 exciton to a lower-energy *n* = 3 exciton, which is not resolved in the steady-state absorption spectrum (Figure 1a) but is clearly observed in the PL spectrum (Figure 1b). To confirm this picture, global analysis of the TA data was performed employing a sequential model (see Supporting Materials for details). The main processes involving the *n* = 2 exciton are characterized by time constants of 260 fs for hot-carrier cooling, 35 ps for energy transfer to the *n* = 3 exciton, and 570 ps for a third component. This last component is attributed to the radiative recombination of the *n* = 2 excitons that do not participate in energy funneling. The good agreement between the fits (solid lines) and the experimental data (open circles) supports the proposed mechanism. The *n* = 1 excitonic feature is not clearly resolved in the TA spectra, likely because of its relatively weak amplitude and partial overlap with the intense photoinduced absorption band centered at 540 nm, which is associated with the *n* = 2 excitonic phase.

**Figure 5:**
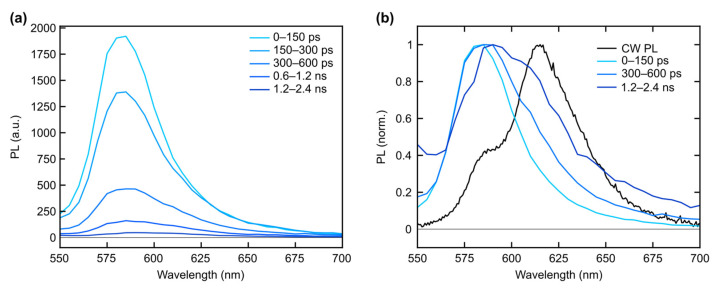
(**a**) Time-resolved photoluminescence spectra of the polycrystalline BAMA film integrated over different time-delay windows. (**b**) Normalized time-resolved photoluminescence spectra of the polycrystalline BAMA film integrated over different time-delay windows (colored solid lines). The continuous-wave (CW) photoluminescence spectrum is shown as a black solid line.

To gain further insight into this long-timescale energy-transfer process, time-resolved emission spectroscopy (TRES) measurements were performed. Figure 5 shows the emission spectra obtained by integrating the signal over different time-delay windows in the spectral region associated with emission from *n* ≥ 2 phases, following excitation at 407 nm with 80 ps diode laser pulses.

In Figure 5a,b, the PL spectra (normalized) recorded following photoexcitation are shown for different time windows. The PL spectra collected within the first hundred picoseconds reveal a broad emission centered at 580 nm corresponding to *n* = 2 exciton emission. As time progresses, the *n* = 2 exciton population undergoes energy transfer to lower-energy excitonic states (*n* ≥ 3) (see Figure S3a for the PL lifetimes). Energy transfer in RP films depends strongly on the spatial arrangement of different n-phases. The *n* = 2 domains are connected to larger-*n* domains and can transfer excitons to lower-energy acceptor phases (*n* ≥ 3), enabling efficient energy funneling. Interestingly, despite the efficient energy transfer, the *n* = 2 population is not completely depleted, as its emission remains visible in the continuous-wave (CW) PL spectrum (Figure 5b).

**Figure 6:**
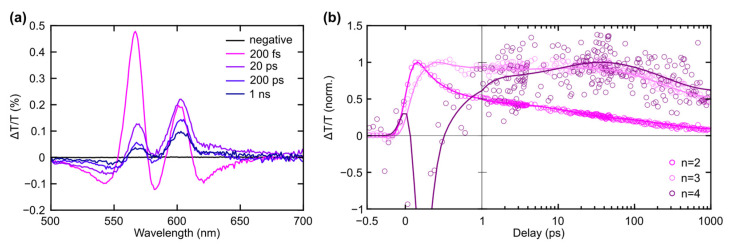
(**a**) TA spectra at different pump-probe delays of the single-crystal BAMA film. (**b**) TA dynamics (open circles) of the single-crystal BAMA film at 570 nm (*n* = 2), 610 nm (*n* = 3), and 630 (*n* = 4). The global fits are shown as solid lines. For clarity, the time axis is displayed on a linear scale up to 1 ps and on a logarithmic scale for longer delay times.

As expected from the steady-state measurements, the TA spectra of the single-crystal BAMA film exhibit a larger number of spectral features associated with *n* ≥ 3 excitonic phases (Figure 6a). Figure 6b shows the TA dynamics of the three positive signals corresponding to the *n* = 2 (570 nm), *n* = 3 (610 nm) and *n* = 4 (630 nm) excitons. Immediately after photoexcitation, an instantaneous bleaching signal appears at 570 nm, together with two photoinduced absorption bands centered at 540 nm and at 590 nm. In contrast to the polycrystalline sample, an additional positive feature centered at approximately 630 nm emerges with a relatively slow rise time of approximately 80 ps. Conversely, the positive signal at 610 nm displays an essentially instantaneous rise, with the apparent delay of approximately 100 fs likely arising from the initial spectral overlap with the negative band centered at 590 nm. Nevertheless, the *n* = 3 exciton population associated with the 610 nm feature seems to continue increasing on the same timescale as the *n* = 4 excitonic signal, suggesting that it is continuously fed by the energy-transfer process. The global analysis provides insight into these underlying dynamics (see the Supporting Materials for details). The main processes involving the *n* = 2 exciton follow a sequence similar to that observed in the polycrystalline sample. An initial 340 fs component is assigned to hot-carrier cooling, followed by a 13 ps component attributed, in this case, to energy transfer to the *n* = 3 and *n* = 4 excitons. Finally, a third, longer component of 260 ps is observed, as in the polycrystalline sample. This component is assigned to the radiative recombination of *n* = 2 excitons that do not participate in the energy-funneling process.

It is worth noting that the global fits (solid lines) show excellent agreement with the experimental data (open circles) in Figure 6b, despite the single-crystal BAMA model containing the same number of kinetic components as that used for the polycrystalline BAMA (Figure 4b). This is particularly significant because the single-crystal BAMA data exhibit an additional signal associated with the *n* = 4 exciton, which is absent in the polycrystalline BAMA data (Figure 4a). These results support the interpretation that the *n* = 2 exciton acts as the main donor state, feeding the two distinct lower-energy excitonic phases, i.e., the *n* = 3 and *n* = 4 phases, through energy-transfer processes occurring on comparable timescales that cannot be disentangled by the sequential model adopted here. Moreover, this analysis provides no evidence of energy transfer from the *n* = 3 to the *n* = 4 exciton. Overall, these observations are consistent with a morphology in which the *n* = 2 phase dominates the excitonic landscape, while higher-n phases are dispersed throughout the film and mainly populated through interphase energy transfer from the *n* = 2 exciton. The absence of this behavior in the polycrystalline sample indicates that the single-crystal-derived growth route promotes a more interconnected phase distribution and a richer exciton-funneling landscape.

**Figure 7:**
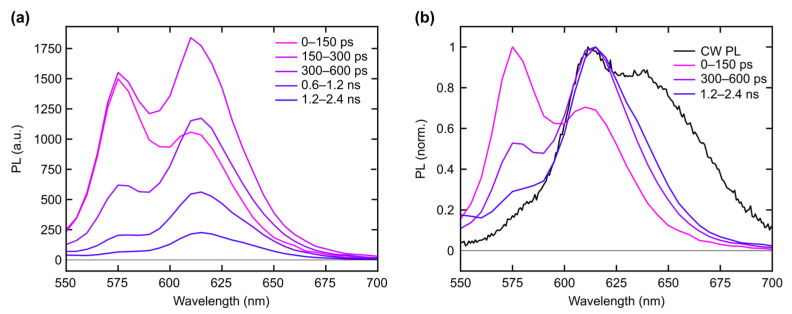
(**a**) Time-resolved photoluminescence spectra of the single-crystal BAMA film integrated over different time-delay windows. (**b**) Normalized time-resolved photoluminescence spectra of the single-crystal BAMA film integrated over different time-delay windows (colored solid lines). The continuous-wave (CW) photoluminescence spectrum is shown as a black solid line.

To complement this analysis, TRES measurements were performed on the single-crystal BAMA film. Figure 7a shows the time-resolved PL spectra recorded over different temporal windows following photoexcitation. The earliest spectra reveal emission from the *n* = 2 exciton at 570 nm and the *n* = 3 exciton at 610 nm. The prompt appearance of the 610 nm signal is expected because of the instantaneous population by the pump as highlighted by the TA measurements. With increasing delay time, the *n* = 2 emission progressively decays, while the lower-energy excitonic phases become increasingly populated, consistent with the exciton-funneling dynamics inferred from TA measurements (see Figure S3b for the PL lifetimes). Consequently, the *n* = 2 contribution is largely suppressed in the CW PL spectrum (Figure 7b), whereas the lowest-energy emissive phase dominates the steady-state emission due to the accumulation of population transferred from the *n* = 2 exciton.

## 3. Materials and Methods

### 3.1. Materials

Lead oxide (PbO, >98%) and lead iodide (>99%) were purchased from Tokyo Chemical Industry Co., Ltd. (Tokyo, Japan). n-butylammonium iodide was purchased from Greatcell Solar Materials Pty Ltd. (Queanbeyan East, Australia). Methylammonium iodide was purchased from Ossila Ltd. (Sheffield, UK). Butylamine (BAI), hydroiodic acid (57 wt % in H_2_O, distilled, stabilized, 99.95%), methylamine hydrochloride (MACL, >98%), heptane, N,N-dimethylformamide (DMF, 99.8%), and hypophosphorous acid solution (H_3_PO_2_, 50 wt % in H_2_O) were purchased from Sigma-Aldrich, Saint Louis, MO, USA. All materials were used as received.

### 3.2. Crystal Synthesis

Crystal synthesis followed the procedure reported by Sidhik et al. to synthesize BA_2_MAPb_2_I_7_ (*n* = 2) [37]. PbO powder (2232 mg) was dissolved in a mixture of 57% *w*/*w* aqueous HI solution (15.0 mL) and 50% aqueous H_3_PO_2_ (2 mL) by heating to boiling under constant magnetic stirring for about 5 min, which formed a bright yellow solution. In a separate beaker, BAI (694 μL) was neutralized with HI 57% *w*/*w* (5 mL) in an ice bath. The BAI solution was added to the hot yellow solution under continuous stirring. Subsequent addition of solid MACl (338 mg) to the hot solution initially produced a dark precipitate, which was subsequently dissolved by heating the combined solution. Stirring was discontinued, and the solution was left to cool to room temperature before standing overnight to crystallize, yielding dark-red crystals. The crystals were collected by filtering via the Büchner funnel process. The crystals were rinsed twice with heptane in the funnel. The crystals were transferred into a vacuum dry oven at ca 70 °C overnight. The formula weight used was 1483.1.

### 3.3. Polycrystalline Solution Preparation

For *n* = 2, MA-based (BA_2_MAPb_2_I_7_): 0.3 mmol of PbI2 (138.4 mg), 0.3 mmol of nBAI (60.4 mg) and 0.15 mmol of MAI (23.9 mg) were dissolved in DMF solvent and stirred overnight.

### 3.4. Film Processing

Quartz substrates were cleaned by sequential sonication in diluted Hellmanex solution (~1% in deionized water), deionized water, acetone, and isopropanol. The substrates were UV ozone treated for 20 min immediately prior to thin film processing. All thin film processing was performed within a nitrogen-filled glovebox. A 0.3 M solution of each of the quasi-2D RP single-crystal perovskites was prepared in DMF solvent. A single-crystal BA_2_MAPb_2_I_7_ perovskite solution was spun at 5000 rpm for 30 s. A polycrystalline BA_2_MAPb_2_I_7_ perovskite solution was spun at 6000 rpm for 30 s. All BA_2_MAPb_2_I_7_ films were annealed at 100 °C for 10 min. To isolate the influence of the formation routes, all films were deposited on identical quartz substrates. Quartz was selected because it is the most suitable substrate for pump–probe measurements, owing to its high transparency at the excitation wavelength (400 nm). Consequently, the observed differences are attributed primarily to variations in the crystallization pathway rather than substrate-dependent effects.

### 3.5. Steady-State Absorption Spectroscopy

The absorbance of the perovskite films was investigated using ultraviolet-visible transmission spectroscopy. Measurements were performed over the 400–900 nm spectral range using a FluoroMax-4 fluorometer (HORIBA, Kyoto, Japan). The absorbance was plotted using the formula A = −log10(I/I_0_), where I_0_ and I are the incident and transmitted light intensity through the sample, respectively.

### 3.6. Atomic Force Microscopy

Measurements were performed in air using an Asylum Research MFP-3D system with Budget Sensors Multi75E-G probes (Sofia, Bulgaria). The cantilevers had spring constants between 2.17 and 2.56 nN/nm. The initial SUM signal, obtained after mounting and aligning each cantilever, ranged from 1.54 to 1.60. A free amplitude of 25 nm with a setpoint of 20 nm was applied for single-crystal-derived BA_2_MAPb_2_I_7_ films. For polycrystalline BA_2_MAPb_2_I_7_ films, a free amplitude of 100 nm and a setpoint of 83 nm were applied. Images were acquired at a scan rate between 0.35 and 0.5 Hz and recorded at 512 × 512-pixel resolution. Post-image processing was carried out in Gwyddion (https://gwyddion.net/, accessed on 26 July 2026) using plane leveling and median of differences correction.

### 3.7. X-Ray Diffraction

Measurements were performed at room temperature using a PANalytical (Malvern, UK) X’Pert^3^ Powder diffractometer operating in Bragg–Brentano reflection geometry. The instrument was equipped with a Cu line-focus X-ray tube operated at an accelerating voltage of 45 kV and a tube current of 40 mA. A Ni filter was used to suppress Cu Kβ radiation, while the incident beam comprised the Cu Kα doublet with wavelengths of λ(Kα_1_) = 1.540598 Å and λ(Kα_2_) = 1.544426 Å, corresponding to an intensity ratio of Kα_2_/Kα_1_ = 0.50. An anti-scatter slit of 1/8° was employed during data acquisition. Diffraction patterns were collected over a 2θ range of 2–45°.

### 3.8. Steady-State PL Spectroscopy

The steady-state PL measurements of the BA_2_MAPb_2_I_7_ perovskite films were performed using the integrating sphere system calibrated with an HL-3P-INT-CAL reference lamp (Ocean Insight, Orlando, FL, USA). A 405 nm continuous-wave diode laser (MDL-III-405-50mW, Class 3B, M^2^ = 1.5) was used as the excitation source. The excitation beam profile at the sample position was measured using a Thorlabs BC106N-VIS/M beam profiler (Newton, NJ, USA), giving an elliptical beam diameter of approximately 0.81 × 0.88 mm (4σ). The corresponding spot area was calculated to be 0.0056 cm^2^ and used to determine the excitation power density. The laser excitation power density was 46.11 mW cm^−2^, corresponding to approximately 1-sun-equivalent excitation conditions. The emitted photoluminescence was collected and analyzed using a grating spectrometer coupled to a CCD detector.

### 3.9. Film Thicknesses

Measurements were performed across multiple sample regions of each sample using a Bruker (Billerica, MA, USA) Dektak XT stylus profilometer. Step edges reaching the substrate were created at distinct surface locations by razor blade scribing. Linear profiles were recorded across each scratch boundary using a 12.5 μm radius stylus under a constant load of 3 mg. The resulting 1D scans were baseline-corrected in Bruker Vision64 software, and local thicknesses were extracted from the corresponding step-height differentials. The software information is available from Bruker at https://www.bruker.com/en/products-and-solutions/test-and-measurement/3d-optical-profilers/vision64-software.html (accessed on 26 July 2026).

### 3.10. Femtosecond Transient Absorption Spectroscopy

The ultrafast spectroscopy setup was fed by a 150 fs, 2 kHz repetition rate Ti: sapphire system (Libra, Coherent, Santa Clara, CA, USA) with a central wavelength of 800 nm. Transient-absorption measurements were performed by pumping at 400 nm (3 μJ cm^−2^) with the second harmonic of the laser output, generated with a 1 mm Type I β-barium borate crystal. The probe pulses, with a spectrum spanning from 450 to 750 nm, were obtained by white-light generation in a 3 mm thick sapphire crystal. The measurements were performed in transmission, and the probe spectrum was detected using a SP2150 Acton, Princeton Instruments spectrometer (Trenton, NJ, USA). The pump beam was modulated by a mechanical chopper at a 1 kHz frequency, and the differential transmission (ΔT/T) spectrum of the probe was measured as a function of probe wavelength and pump–probe delay.

### 3.11. Time-Resolved Time-Correlated Single Photon Counting (TCSPC)

Measurements are performed with a NanoLog composed of an iH320 spectrograph and PPD-850 single-photon detector module by exciting at 407 nm with a DeltaTime series DD-405L Delta Diode Laser HORIBA Italia S.r.l., Milano, Italy.

## 4. Conclusions

In this work, we compared BA_2_MAPb_2_I_7_ quasi-2D Ruddlesden–Popper (RP) perovskite thin films fabricated through a single-crystal-derived route with films prepared by a conventional polycrystalline route. We demonstrate that the two fabrication approaches significantly affect the film morphology as well as their steady-state and transient optical properties, including photoluminescence and transient absorption responses.

Time-resolved emission spectra and pump–probe measurements reveal markedly different exciton dynamics in the two systems. While the polycrystalline sample is mainly composed of *n* = 2 and *n* = 3 phases and exhibits limited interphase energy-transfer pathways, the single-crystal-derived film displays a richer excitonic landscape characterized by the presence of higher-(n) phases. The temporal evolution of the photobleaching features shows that the *n* = 2 exciton acts as the primary donor state, transferring its population toward lower-energy excitonic phases through multiple channels operating on distinct timescales. The close correspondence between the decay kinetics of the *n* = 2 excitonic feature and the rise in lower-energy photobleaching signals provides direct evidence of exciton funneling across the phase distribution. In the single-crystal-derived film, the coexistence of transfer processes points to a heterogeneous network of interconnected excitonic domains, where the predominant *n* = 2 phase feeds a smaller population of higher-(*n*) phases. These observations indicate that the excitonic landscape of the single-crystal-derived film is governed by efficient interphase energy transfer and highlight the central role of the *n* = 2 phase in mediating exciton migration toward lower-bandgap domains.

Overall, our results demonstrate that the synthetic route can strongly influence the phase distribution and exciton-transport dynamics in RP perovskites. Controlling these parameters provides an effective strategy for tailoring exciton funneling and energy-transfer processes in low-dimensional perovskite heterostructures.

## Data Availability

The data presented in this study are available through the ORDA repository at the DOI: 10.15131/shef.data.32923832.

## Supplementary Materials

The following supporting information can be downloaded at: https://www.mdpi.com/article/10.3390/molecules31152636/s1, Figure S1: XRD plot of normalized patterns of the polycrystalline (PC) and single-crystal-derived (SC) BA_2_MAPb_2_I_7_ films; Figure S2: Magnified XRD plot of polycrystalline BA_2_MAPb_2_I_7_ thin film; Figure S3: PL time decays for the (a) polycrystalline and (b) single-crystal BAMA sample at 580 nm (*n* = 2), 610 nm (*n* = 3) and 640 nm (*n* = 4). The time axis is displayed on a linear scale until 1000 ps and on a logarithmic scale onwards; Figure S4: EAS of the polycrystalline BA_2_MAPb_2_I_7_; Figure S5: EAS of the single-crystal BA_2_MAPb_2_I_7_; Figure S6: Residual map obtained from the global fit of the polycrystalline BA_2_MAPb_2_I_7_; Figure S7: Residual map obtained from the global fit of the single-crystal BA_2_MAPb_2_I_7_; Table S1: Absorption peaks for the PC and SC BA_2_MAPb_2_I_7_ films; Table S2: XRD peak indexing and comparison between experimental thin film data and simulated reference. Note S1: Global Fitting of Transient Absorption. Refs. [14,65,66] are cited in the Supplementary Materials.
