# Supplementary material for: Crystal-Growth-Controlled Exciton Funneling in BA2MAPb2I7 Ruddlesden–Popper Perovskite Thin Films
Source: Molecules. 2026 Jul 29;31(15):2636. doi: 10.3390/molecules31152636 (PMC13468545; doi:10.3390/molecules31152636)
Supplement: Supplementary file 1 [file molecules-31-02636-s001.zip › molecules-4455797-supplementary.pdf]

Supplementary Materials

Crystal-Growth-Controlled Exciton Funneling in $BA_2MAPb_2I_7$ Ruddlesden–Popper Perovskite Thin Films

Grace Dansoa Tabi [1,†], Diego Florio [2,3,†], Chiara Botta [4], Alexandra J. Ramadan [1,*] and Tersilla Virgili [3,*]

[1] School of Mathematical and Physical Sciences, University of Sheffield, Hounsfield Road, Sheffield S3 7RH, UK; g.d.tabi@sheffield.ac.uk
[2] Istituto di Fotonica e Nanotecnologia—National Research Council (CNR), Piazza Leonardo da Vinci 32, 20133 Milan, Italy; diego.florio@polimi.it
[3] Dipartimento di Fisica, Politecnico di Milano, Piazza Leonardo da Vinci 32, 20133 Milan, Italy
[4] Istituto di Scienze e Tecnologie Chimiche "Giulio Natta"—National Research Council (CNR), Via A. Corti 12, 20133 Milan, Italy; chiara.botta@scitec.cnr.it
* Correspondence: a.ramadan@sheffield.ac.uk (A.J.R.); tersilla.virgili@cnr.it (T.V.)
† These contributed equally to the work.

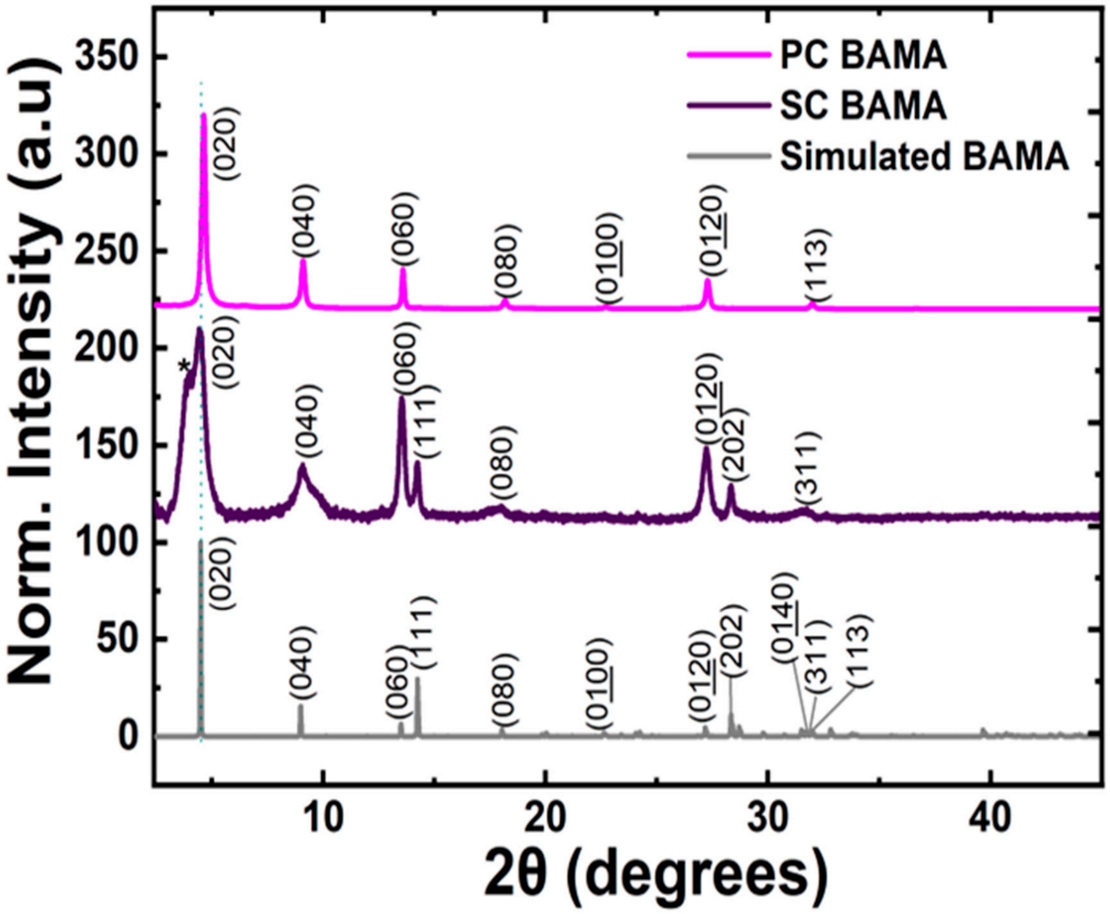

Figure S1: XRD plot of normalised patterns of the polycrystalline (PC) and single-crystal derived $BA_2MAPb_2I_7$ thin films compared with the simulated reference CCDC 1478376 [1]. The asterisk is a non-identified phase.

Table S1. Absorption profiles of the *n* = 2 $BA_2MAPb_2I_7$ perovskite thin films.

Material System	Main Peaks (nm)	Secondary Peaks (nm)
SC $BA_2MAPb_2I_7$	567	600 (n =3)
PC $BA_2MAPb_2I_7$	569	512 (n =1)

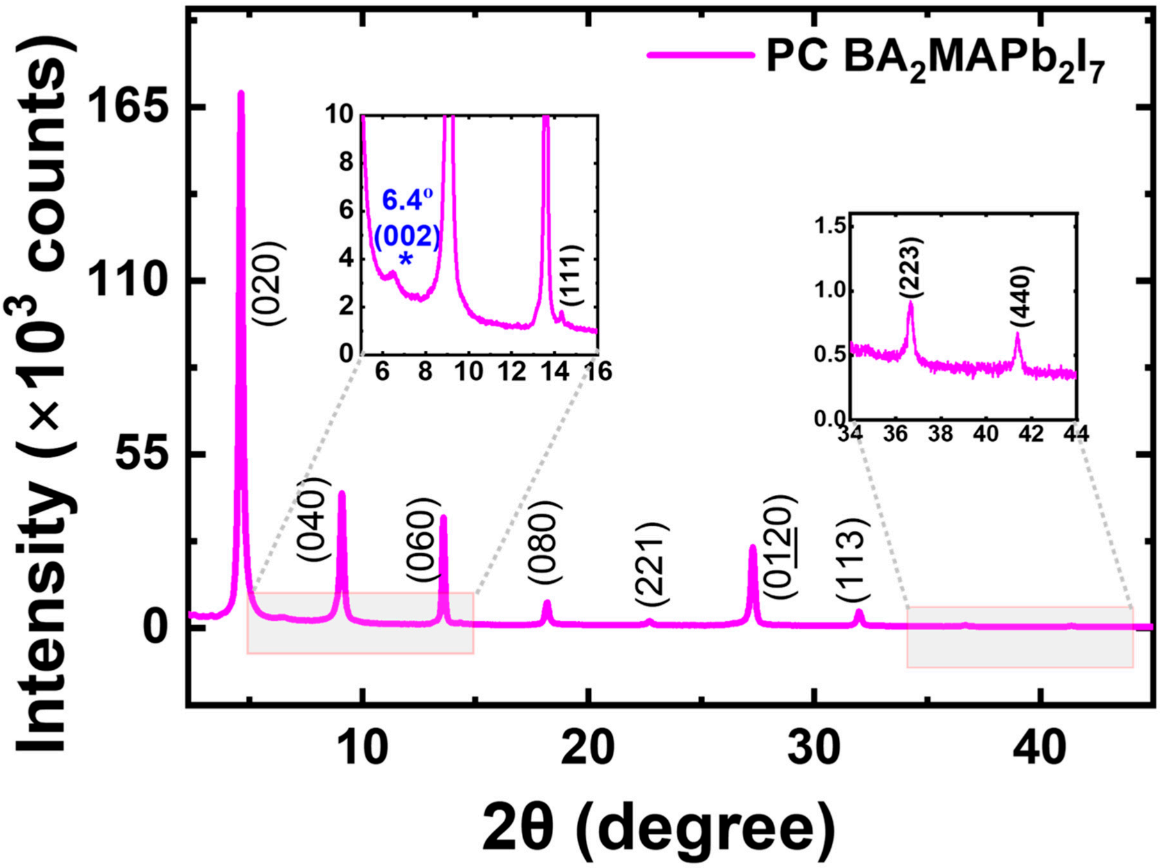

Figure S2: XRD plot of the polycrystalline $BA_2MAPb_2I_7$ thin film assigned by comparing experimental data with the simulated reference CCDC 1478376 [1] and CCDC 1268429 [2]. The inset shows a magnified view of the highlighted 2θ regions to clearly resolve the weak diffraction patterns.

In the polycrystalline $BA_2MAPb_2I_7$ thin film, the presence of weak reflection near 6.4° 2θ, assigned to (002) plane of the n = 1 phase (marked by asterisk). This assigned peak is consistent with the *n* = 1 excitonic absorption feature in Figure 1a, indicating the presence of minor *n* = 1 phase fraction in the predominantly *n* = 2 polycrystalline $BA_2MAPb_2I_7$ film.

Table S2. XRD peak indexing and comparison between experimental thin film data and simulated reference CCDC 1478376 [1] for SC and PC $BA_2MAPb_2I_7$ thin films. X-ray sources used were Cu - $K_{\alpha1}$ and -$K_{\alpha2}$ in the ratio of 0.5. Blank entries indicate diffraction patterns that were not observed experimentally and therefore could not be assigned.

SC $BA_2MAPb_2I_7$	PC $BA_2MAPb_2I_7$	Simulated $BA_2MAPb_2I_7$ (CCDC 1478376) [1]	Miller Index
2θ (°)	2θ (°)	2θ (°)	(hkl)
4.45	4.60	4.49	(020)
9.04	9.10	9.00	(040)
13.53	13.59	13.52	(060)
14.22	14.23	14.24	(111)
18.00	-	18.02	(080)
-	18.15	18.07	(080)
-	22.72	22.70	(221)
27.20	27.29	27.24	(0<u>12</u>0)
28.33	-	28.33	(202)
31.74	-	31.71	(311)
-	31.97	31.97	(113)
-	36.70	36.72	(223)
-	41.38	41.36	(440)

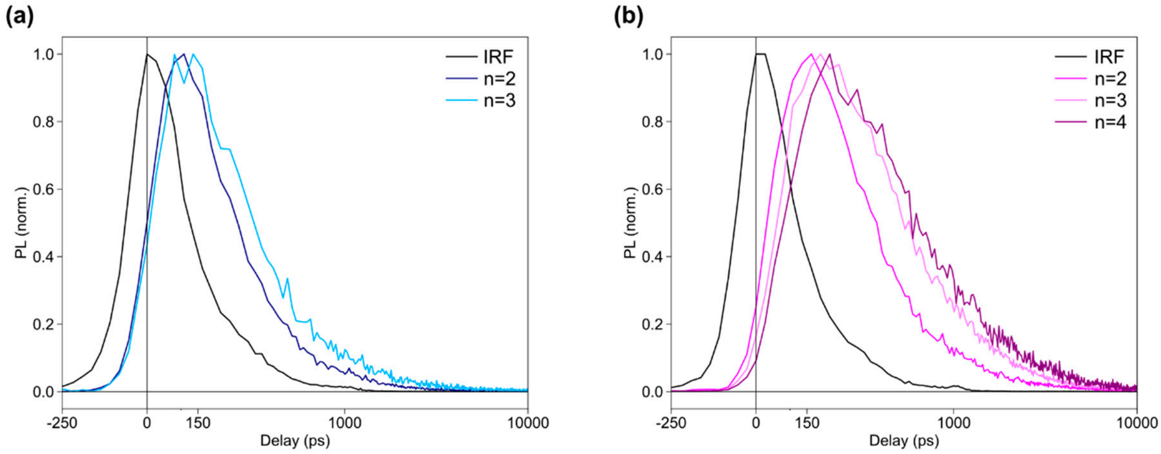

Figure S3: Normalised PL lifetimes of the (a) polycrystalline and (b) single crystal $BA_2MAPb_2I_7$ thin films at 580 nm (*n* = 2), 610 nm (*n* = 3) and 640 nm (*n* = 4). The instrument response function (IRF) of the experiment is also shown in black. The time axis is displayed on a linear scale until 1000 ps and on a logarithmic scale onwards.

Supplementary Note S1 - Global Fitting of Transient Absorption

We globally fitted the TA data of the polycrystalline and single crystal $BA_2MAPb_2I_7$ shown, respectively, in Figure 4 and Figure 6 of the main text, employing a sequential model using Glotaran [3]. In the sequential model, the i-th evolution associated spectrum (EAS) evolves into the (i+1)-th EAS with a time constant τ_i. The EAS labeled "Last" represents the TA spectrum remaining at approximately 1 ns delay time.

Figure S4 shows the EAS obtained from the fit of the polycrystalline $BA_2MAPb_2I_7$. Three kinetic components are needed to adequately fit the data (Root Mean Squared Error (RMSE) = 2.07×10^{-4}). We attribute the first component ($\tau_1 = 260 \pm 4\, fs$) mainly to hot carrier cooling. The second component ($\tau_2 = 35 \pm 1\, ps$) is attributed predominantly to energy transfer from the *n* = 2 exciton to the *n* = 3 exciton. Compared with the EAS associated to τ_3 (blue solid line), the EAS associated to τ_2 (red solid line) exhibits a lower amplitude of the *n* = 2 excitonic feature and a higher amplitude of the *n* = 3 excitonic feature. This redistribution of spectral weight is consistent with the proposed energy-transfer process. Finally, the third component ($\tau_3 = 570 \pm 17\, ps$) represents a decay process common to both exciton species.

Figure S5 shows the EAS obtained from the fit of the single crystal $BA_2MAPb_2I_7$. The analysis of the EAS is similar to the polycrystalline counterpart with a new exciton state corresponding to *n* = 4 phase (Root Mean Squared Error (RMSE) = 2.09×10^{-4}). The main differences are an instantaneous population of the *n* = 3 exciton upon excitation and a faster energy transfer from the *n* = 2 to the *n* = 3 and *n* = 4 exciton ($\tau_2 = 13 \pm 1\, ps$), which results in the transfer of most of the exciton population to the *n* ≥ 2 state.

Figures S6 and S7 show the residual maps obtained from the global fits of the polycrystalline and single crystal $BA_2MAPb_2I_7$ thin films, respectively. No pronounced systematic spectral or temporal structure is observed in the residuals, supporting the adequacy of the kinetic models within the signal-to-noise ratio of the measurements.

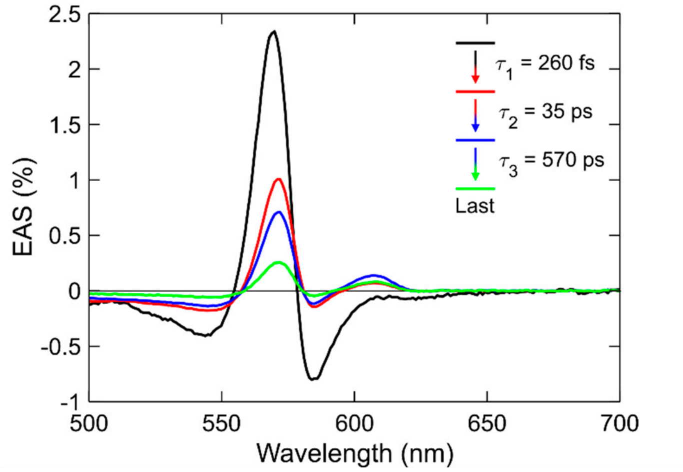

Figure S4. EAS of the polycrystalline $BA_2MAPb_2I_7$ thin film.

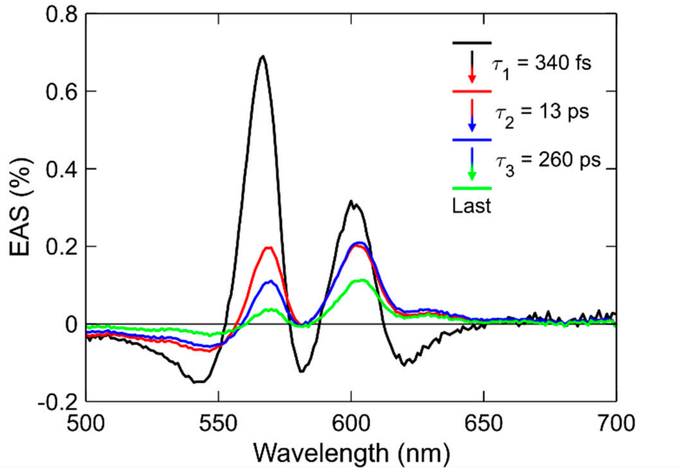

Figure S5. EAS of the single-crystal $BA_2MAPb_2I_7$ thin film.

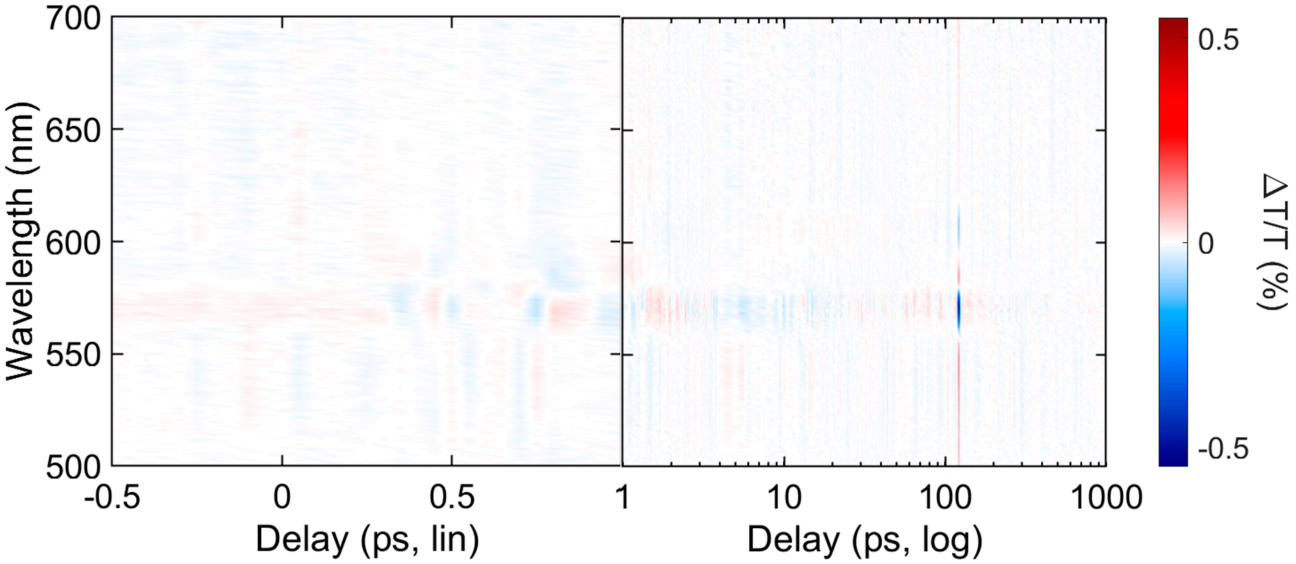

Figure S6. Residual map obtained from the global fit of the polycrystalline $BA_2MAPb_2I_7$ thin film.

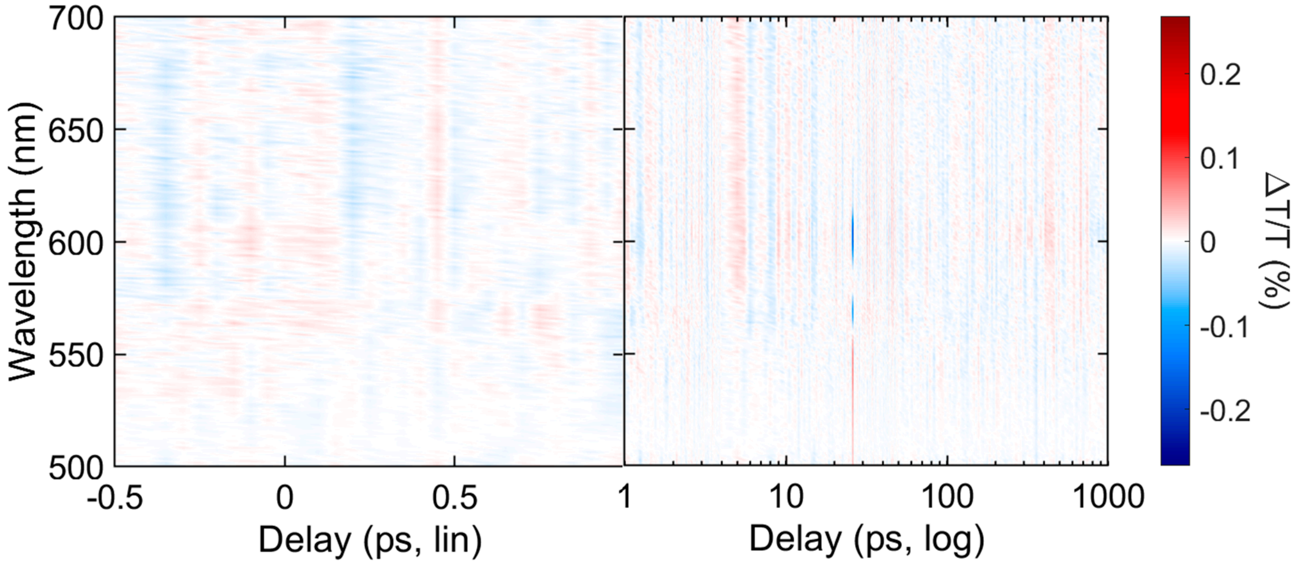

Figure S7. Residual map obtained from the global fit of the single-crystal $BA_2MAPb_2I_7$ thin film.

References

1. Stoumpos, C. C. *et al.* Ruddlesden–Popper Hybrid Lead Iodide Perovskite 2 Homologous Semiconductors. *Chem. Mater.* **28**, 2852–2867 (2016).

2. Mitzi, D. B. Synthesis, Crystal Structure, and Optical and Thermal Properties of $(C_4H_9NH_3)_2MI_4$ (M = Ge, Sn, Pb). *Chem. Mater.* **8**, 791–800 (1996).

3. J. J. Snellenburg, S. Laptenok, R. Seger, K. M. Mullen, I. H. M. van Stokkum, Glotaran: A Java-Based Graphical User Interface for the R Package TIMP. *Journal of Statistical Software* **49**, 1 - 22 (2012)
